# Whole-Cell or Acellular Pertussis Primary Immunizations in Infancy Determines Adolescent Cellular Immune Profiles

**DOI:** 10.3389/fimmu.2018.00051

**Published:** 2018-01-24

**Authors:** Saskia van der Lee, Lotte H. Hendrikx, Elisabeth A. M. Sanders, Guy A. M. Berbers, Anne-Marie Buisman

**Affiliations:** ^1^Centre for Infectious Disease Control, National Institute for Public Health and the Environment (RIVM), Bilthoven, Netherlands; ^2^Department of Paediatric Immunology and Infectious Diseases, Wilhelmina Children’s Hospital, University Medical Centre, Utrecht, Netherlands; ^3^Research Centre Linnaeus Institute, Spaarne Hospital, Hoofddorp, Netherlands

**Keywords:** immune profiles, (pre-)adolescents, T-helper 1/Th2 ratio, pertussis, infant priming

## Abstract

**Introduction:**

Pertussis is re-emerging worldwide, despite effective immunization programs for infants and children. Epidemiological studies show a more limited duration of protection against clinical pertussis in adolescents primed with acellular pertussis (aP) vaccines during infancy than those who have been primed with whole-cell pertussis (wP) vaccines. This study aimed to determine whether memory immune responses to aP, diphtheria, and tetanus vaccine antigens following booster vaccinations at 4 and 9 years of age differ between wP- versus aP-primed children.

**Methods:**

In a cross-sectional study, blood was collected of DTwP- or diphtheria, tetanus, and aP (DTaP)-primed children before, 1 month, and 2 years after the preschool DTaP booster administered at 4 years of age (*n* = 41–63 per time point). In a longitudinal study, blood was sampled of DTwP- or DTaP-primed children before, 1 month, and 1 year after a preadolescent Tdap booster at 9 years of age (*n* = 79–83 per time point). Pertussis, diphtheria, and tetanus vaccine antigen-specific IgG levels, B-cell and T-cell responses were determined.

**Results:**

After the preschool booster vaccination, IgG levels were significantly higher in aP-primed as compared with wP-primed children until 6 years of age. Before the preadolescent Tdap booster vaccination, humoral and cellular immune responses were similar in aP- and wP-primed children. However, the Tdap booster vaccination induced lower vaccine antigen-specific humoral, B-cell, and T-helper 1 (Th1) cell responses resulting in significantly lower Th1/Th2 ratios in aP-primed compared with wP-primed children.

**Conclusion:**

The memory immune profiles at preadolescent age to all DTaP vaccine antigens are already determined by the wP or aP combination vaccines given in infancy, showing a beneficial Th1-dominated response after wP-priming. These immunological data corroborate epidemiological data showing that DTaP-primed adolescents are less protected against clinical pertussis than DTwP-primed children.

## Introduction

Since the introduction of whole-cell pertussis (wP) vaccines in the 1940s, widespread vaccination of children strongly reduced the incidence of clinical pertussis (1). Despite effective infant immunization programs and high vaccination coverage in developed countries, pertussis is re-emerging worldwide, not only in non- or partially vaccinated infants but also in adolescents and adults (2–8). Estimates from the WHO suggest that approximately 16 million cases and 200,000 deaths are due to pertussis annually (9). Large population-based serosurveillance studies conducted in the Netherlands reported up to 9% of asymptomatic pertussis infections in the population above 9 years of age (10, 11).

In a response to the high number of adverse reactions after wP vaccination, acellular pertussis (aP) vaccines have been developed and implemented in the USA from the mid 1990s and subsequently in many national immunization programs (NIP) of high-income countries in the following decades (12). In the Netherlands, the wP vaccine was replaced by the aP vaccines for the infant priming vaccination series in 2005. The switch from wP to aP vaccines did not cease pertussis re-emergence but rather contributed to the increased incidence of pertussis. Cyclic outbreaks of pertussis are being reported regularly, with the epidemic of 2012 being the largest one since pertussis vaccine introduction (6, 13). The reason of this resurgence is multifactorial, with improved diagnostics, enhanced surveillance, and the switch from wP to aP vaccines but also changes in genetic composition of pertussis strains and especially rapidly waning immunity after vaccination or even after natural infection. Epidemiological studies conducted in the USA and Australia indicated that preschool (at age 4–6 years) and adolescent (at age 11–13 years) aP booster vaccinations only protect for a few years against clinical pertussis, and that vaccine acquired protection wanes more rapidly in individuals primed with aP vaccines at infancy than individuals who were primed with wP vaccines at infancy (14–16). Furthermore, another study also indicated that the increased clinical pertussis incidence in aP-primed adolescents was not cofounded by age or time since last booster vaccination (17). This means that significant numbers of aP-primed individuals become susceptible to infection within a few years after a booster vaccination. Pertussis vaccine studies in the baboon model have demonstrated that aP vaccines prevent clinical disease, but do not preclude asymptomatic infection, colonization, and transmission, which is associated with a lack of T-helper 1 (Th1) immune responses (18).

The immune mechanisms important for protection against pertussis in humans still remain elusive. Protection is suggested to be mediated by both humoral and cellular immunity (19, 20). Higher pertussis-specific antibody levels and memory B-cell responses have been reported in aP-primed versus wP-primed children after a diphtheria, tetanus, and aP (DTaP) booster at age 4 years (21, 22). In addition, DTaP booster vaccination induced higher Th1 and Th2 T-cell responses in preschool aP-primed children compared with wP-primed children (23). Th1 cells are crucial for bacterial clearance and therefore more associated with protection against pertussis than Th2 cells (24).

To elucidate the immune mechanism in relation to long-term protection against pertussis, it is important to evaluate pertussis-specific immune responses over time. In this study, long-term humoral and cellular immune responses to pertussis have been determined in groups of children till adolescent age who received either DTwP or DTaP combination vaccines in infancy following two successive pertussis booster vaccinations at the age of 4 and 9 years.

## Methods

### Study Design and Participants

**Figure 1:**
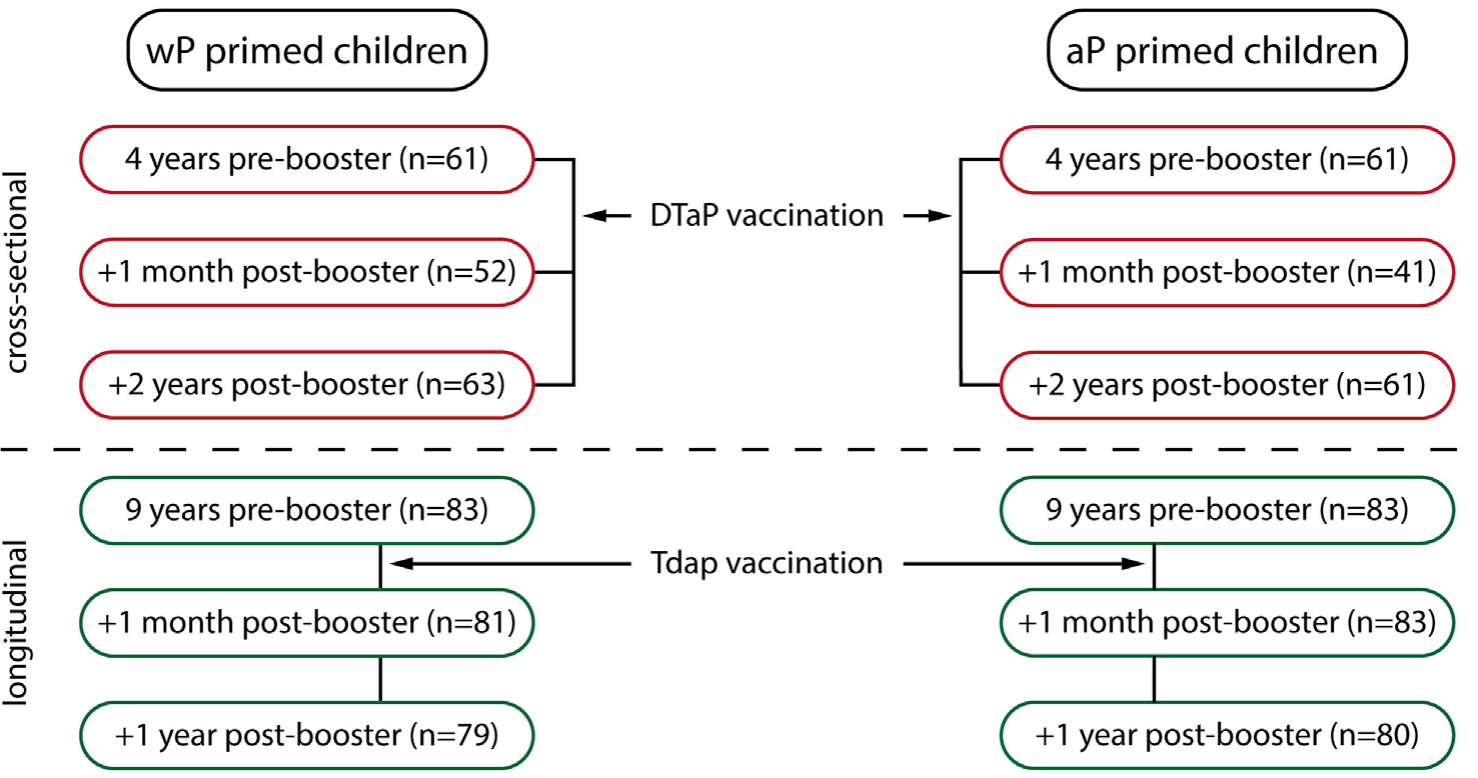
Overview of the study groups. Schematic overview of the six blood sampling time points. Children were primed with either whole-cell pertussis (wP) or acellular pertussis (aP) combination vaccines in the first year of life (2, 3, 4, and 11 months of age) and received a diphtheria, tetanus, and aP (DTaP) booster vaccination at 4 years of age, all according to the Dutch national immunization program. Groups of children were sampled cross-sectionally pre-booster, 1 month and 2 years (at 6 years of age) post DTaP booster vaccination at 4 years of age (indicated in red) (25). In addition, children 9 years of age, primed with either wP vaccines (26, 27) or with aP vaccines (NTR4089, Figure E1 and Method S1 in Supplementary Material) received an additional Tdap booster vaccination and were sampled longitudinally before, 1 month, and 1 year after the booster (indicated in green).

For this study, blood samples were collected at six different time points from children primed with either wP or aP combination vaccines in the first year of life (Figure 1). The participants received their vaccinations according to the Dutch NIP: DTwP or DTaP at 2, 3, 4, and 11 months of age and DTaP at 4 years of age. Blood was cross-sectionally sampled before the preschool DTaP booster vaccination at age 4 years, and 1 month and 2 years after the preschool booster. After an additional Tdap booster vaccination at 9 years of age, blood was longitudinally sampled before, 1 month, and 1 year from groups of wP- or aP-primed children. This aP booster is not implemented in the Dutch NIP, therefore, these children participated in a longitudinal phase IV intervention study. All information regarding study recruitment, characteristics, and flow charts were previously described [ISRCTN65428640 (25) and ISRCTN64117538 (26, 27)], except for the aP-primed children 9 years of age. Information regarding the recruitment and flow chart of these aP-primed 9-year olds is given in Method S1 and Figure E1 in Supplementary Material (2013-001864-50^1^; NTR4089^2^). Sex distribution between the wP-primed and aP-primed groups was similar at all time points. For all participants, written informed consent was obtained from both parents and/or legal representatives in accordance with the Declaration of Helsinki.

### Vaccination Background

During infancy, children received either a diphtheria, tetanus, wP, inactivated polio virus, *Haemophilus influenzae* type b (DTwP-IPV-Hib; Netherlands Vaccine Institute, Bilthoven, the Netherlands) (wP-primed children), or a DTaP-IPV-Hib [Infanrix-IPV-Hib™, GlaxoSmithKline (GSK), Rixensart, Belgium] (aP-primed children) combination vaccine at 2, 3, 4, and 11 months of age. Children received a pediatric DTaP booster vaccination at 4 years of age [Infanrix-IPV™, GSK; containing 25 µg pertussis toxin (PT) and filamentous hemagglutinin (FHA), 8 µg pertactin (Prn), ≥30 IU diphtheria toxoid (Dd), and ≥40 IU tetanus toxoid (Td)]. In addition, a Tdap booster vaccination was administered to children 9 years of age (Boostrix-IPV™, GSK; containing 8 µg PT and FHA, 2.5 µg Prn, ≥2 IU Dd, and ≥20 IU Td).

### Blood Samples

Blood was sampled at 4 years of age, before the DTaP booster vaccination, 1 month (28 ± 2 days), and 2 years (±2 weeks) after the DTaP booster. From children 9 years of age, blood was sampled just before, 1 month (28 ± 2 days), and 1 year (±2 weeks) after a Tdap booster vaccination. Vacutainer cell preparation tubes containing sodium citrate (Becton Dickinson Biosciences, San Diego, CA, USA) were used for all blood samplings. Peripheral blood mononuclear cells were isolated within 18 h and stored at −135°C, and plasma was stored at −20°C as described (28).

### Serological Analysis

Plasma IgG antibody concentrations against PT, FHA, Prn, Dd, and tetanus toxin were quantified using the fluorescent bead-based multiplex immunoassay as described (2, 29). The WHO international standard (Pertussis Antiserum first international standard, 06/140, NIBSC, Potters Bar, UK) was used to express pertussis IgG concentrations in IU/mL.

### Memory B- and T-Cell Responses

From a randomly selected subset of 20 longitudinal blood samples of the children aged 9 years, PT, FHA, Prn, and Td-specific enzyme-linked immunospot assays were performed on purified B-cells to determine the numbers of antigen-specific IgG producing memory B-cells (22, 28). The same PBMC samples, depleted of CD19^+^ cells, were stimulated for 5 days with PT (heat inactivated), FHA, Prn, Td, or pokeweed mitogen and culture supernatants were stored at −80°C (23). In these supernatants, the cytokines interferon-gamma (IFN-γ) (Th1), interleukin-13 (IL-13) (Th2), IL-17 (Th17), and IL-10 (Treg) were quantified using the Bio-Plex cytokine assay kits (Bio-Rad Laboratories, Hercules, CA, USA) (27).

### Statistical Analyses

Geometric mean concentrations with corresponding 95% confidence intervals were calculated for antigen-specific IgG and cytokine responses. Numbers of antigen-specific memory B-cells were counted per 10^5^ B-cells and corresponding geometric mean values were calculated. Normal distribution of (log-transformed) data was checked before analysis. Differences in IgG concentrations between independent or paired samples were tested with corresponding *t*-tests. Differences in not normally distributed B-cell numbers and cytokine concentrations were tested with Mann–Whitney *U* (independent samples) or Wilcoxon signed ranks (paired samples) tests. Differences in proportion of children with a PT-IgG concentration ≥50 IU/mL were tested with Chi-square tests. Within the longitudinal studies, *p*-values were corrected for multiple testing according to the Bonferroni test. *p*-Value <0.05 was considered statistically significant. Data were analyzed using GraphPad Prism 6 (GraphPad Software, La Jolla, CA, USA), and SPSS statistics 22 (IBM, Armonk, NY, USA).

## Results

### Study Groups

The study characteristics of wP- and aP-primed children 4–6 years of age and wP-primed children 9 years of age have been described previously (25–27). The flow chart of aP-primed children 9 years of age are depicted in Figure E1 in Supplementary Material. A schematic overview of all groups is depicted in Figure 1.

### IgG Antibody Levels after the Preschool DTaP Booster Vaccination in Children 4–9 Years of Age

**Figure 2:**
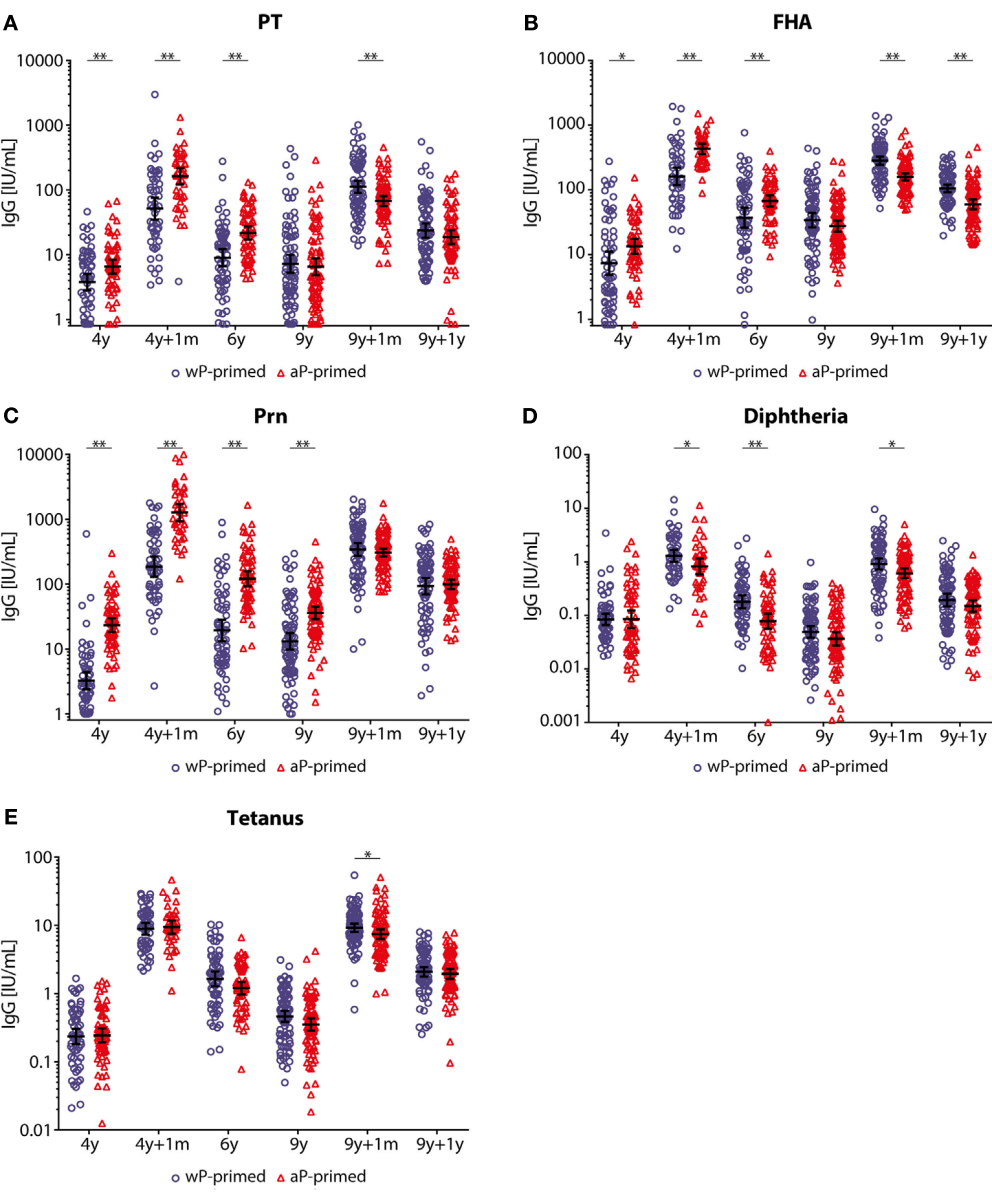
IgG antibody levels in children 4–9 years of age covering two booster vaccinations. **(A)** Pertussis toxin (PT), **(B)** filamentous hemagglutinin (FHA), **(C)** pertactin (Prn), **(D)** diphtheria toxoid, and **(E)** tetanus toxin-specific IgG levels (IU/mL) of 4- to 9-year-old whole-cell pertussis (wP)- (blue circles) or acellular pertussis (aP)-primed (red triangles) children. Blood was sampled cross-sectionally at 4 years of age before, 1 month, and 2 years (6 years of age) after the preschool diphtheria, tetanus, and aP booster and from different children longitudinally at 9 years of age before, 1 month, and 1 year after the preadolescent Tdap booster. Note, black lines indicate geometric mean concentration with 95% confidence interval, *p-value <0.05, and **p-value <0.01. Note, data of PT, FHA, and Prn for wP-primed children aged 4–9 years and aP-primed children aged 4–6 years were previously published (25, 26).

After the preschool DTaP booster, pertussis-specific IgG levels were significantly higher in aP-primed compared with wP-primed children until 6 years of age (Figure 2; Table E1 in Supplementary Material). Moreover, Prn-IgG levels remained significantly higher in aP-primed children until 9 years of age (Figure 2). In contrast, diphtheria-IgG levels were significantly lower until 6 years of age in aP-primed as compared with wP-primed children, whereas no differences were observed for tetanus-IgG levels between the two groups. In general, all pertussis-specific IgG levels were low at 9 years of age, although significantly higher in both groups as compared with preschool booster levels at 4 years of age, with the exception of PT-IgG levels in aP-primed children aged 9 years (Table E1 in Supplementary Material). Diphtheria-IgG levels were significantly lower in both wP- and aP-primed children aged 9 years compared with levels before the preschool booster.

### IgG Antibody Kinetics after a Preadolescent Tdap Booster Vaccination in Children 9 Years of Age

**Figure 3:**
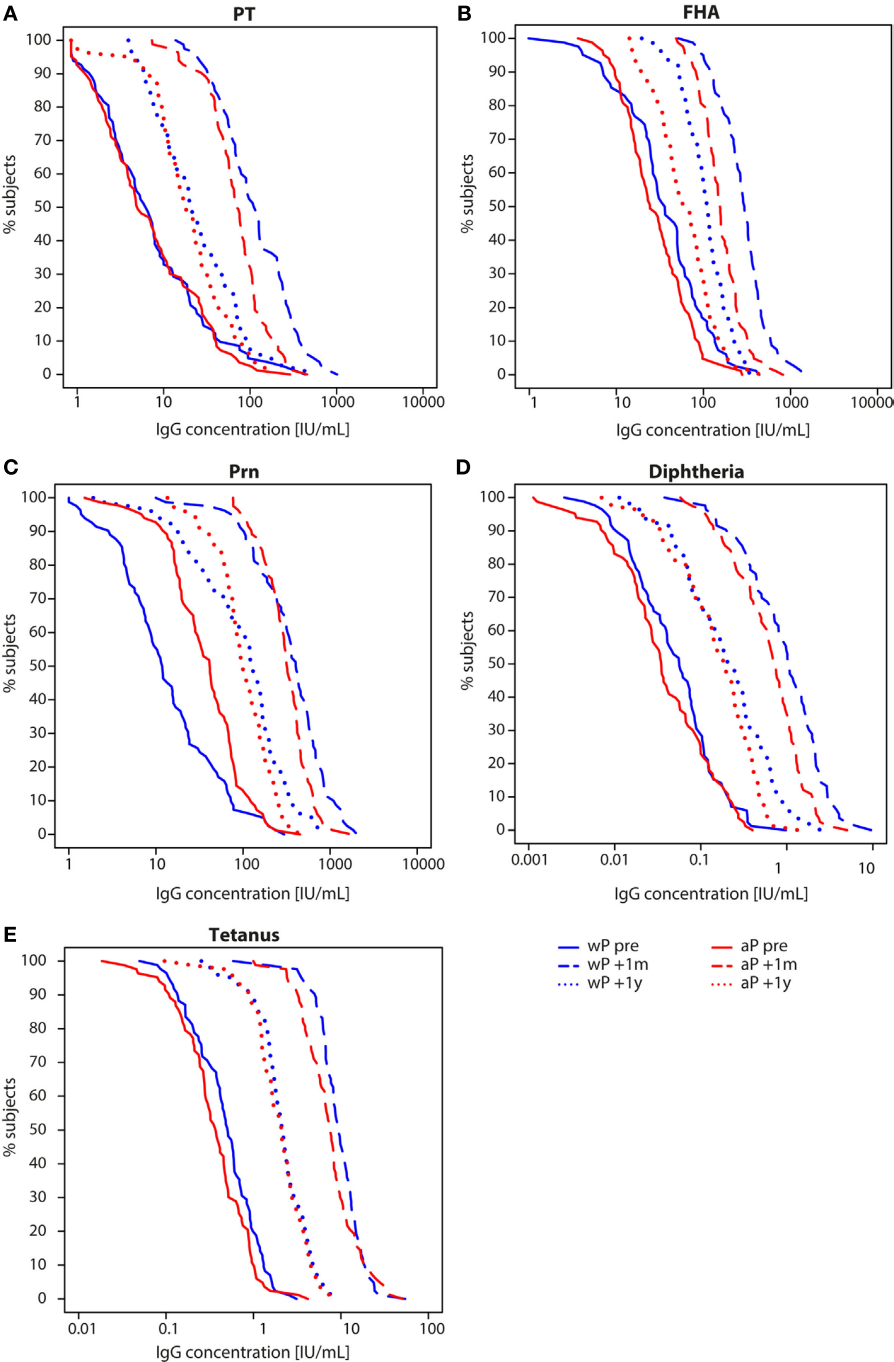
Reverse cumulative distribution curves of IgG antibody levels in children 9 years of age before and after a Tdap booster vaccination. Reverse cumulative distribution curves of **(A)** pertussis toxin (PT), **(B)** filamentous hemagglutinin (FHA), **(C)** pertactin (Prn), **(D)** diphtheria toxoid, and **(E)** tetanus toxin-specific IgG levels of whole-cell pertussis (wP)- (blue) and acellular pertussis-primed (red) children 9 years of age before (solid lines), 1 month (striped lines), and 1 year (dotted lines) after a preadolescent Tdap booster vaccination. Note, data of PT, FHA, and Prn for wP-primed children were previously published (26).

IgG antibody levels specific for all five vaccine antigens (PT, FHA, Prn, diphtheria, and tetanus) were significantly higher 1 month and 1 year after the preadolescent Tdap booster at 9 years of age compared with pre-booster levels for all children (Table E1 in Supplementary Material). However, we observed differences in dynamics between wP- and aP-primed children. One month after the preadolescent Tdap booster, aP-primed children had significantly lower PT-, FHA-, diphtheria-, and tetanus-IgG levels compared with wP-primed children (all *p*-values <0.001), a difference which remained for at least 1 year for FHA-IgG (*p*-value <0.001) (Figures 2 and 3; Table E1 in Supplementary Material). The increase in IgG levels for the three pertussis antigens after the preadolescent booster was significantly less in aP-primed children compared with wP-primed children, while no differences were observed for diphtheria and tetanus (Table E2 in Supplementary Material).

To exclude possible differences in exposure to pertussis between wP- and aP-primed groups, we compared the IgG levels against PT, which is the specific antigen for *Bordetella pertussis*. We found no difference in the proportion of children 9 years of age with a PT-IgG concentration ≥50 IU/mL [suggestive for recent pertussis infection (11, 30)] before the Tdap booster (*p*-value = 0.576) (Figure 3A). None of the children indicated to have had clinical pertussis in the year preceding the preadolescent booster vaccination.

### B-Cell Responses after a Preadolescent Tdap Booster Vaccination in Children 9 Years of Age

**Figure 4:**
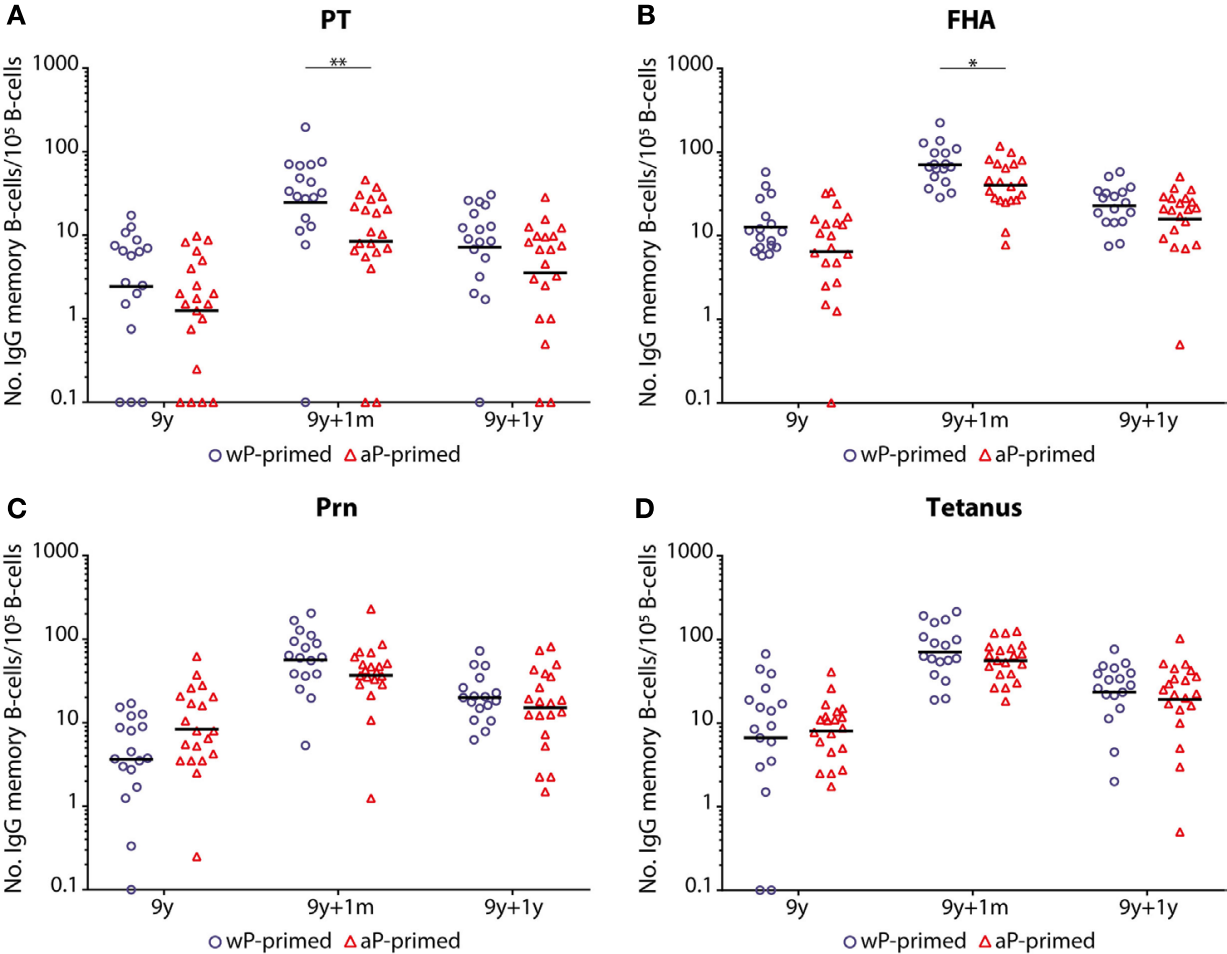
Numbers of memory B-cells in children 9 years of age before and after a Tdap booster vaccination. The numbers of **(A)** pertussis toxin (PT), **(B)** filamentous hemagglutinin (FHA), **(C)** pertactin (Prn), and **(D)** tetanus toxoid-specific IgG producing memory B-cells per 10^5^ IgG producing B-cells of whole-cell pertussis (wP)- (blue circles) or acellular pertussis (aP)-primed (red triangles) children 9 years of age before, 1 month, and 1 year after a preadolescent Tdap booster vaccination. Note, black lines indicate geometric mean numbers, *p-value <0.05, and **p-value <0.01. For all antigens, values of pre versus 1 month, pre versus 1 year, and 1 month versus 1 year were all significantly different within the wP- and aP-primed longitudinal groups of children (all *p*-values <0.05). Note, data of wP-primed children were previously published (26).

No differences were observed in the number of antigen-specific memory B-cells between wP- and aP-primed children before the preadolescent booster (Figure 4). Similar to PT- and FHA-IgG levels, the numbers of PT- and FHA-specific memory B-cells were significantly lower 1 month after the booster in aP- compared with wP-primed children (*p*-value = 0.005 and 0.018, respectively). This trend was also observed for Prn and tetanus, but failed to reach significance. One year after the booster, differences in numbers of memory B-cells had disappeared. Although numbers of Prn-specific memory B-cells were not significantly different, a significant smaller increase in numbers between pre (T0) and 1 month post-booster (T1) vaccination was observed in aP- versus wP-primed children (GM T1/T0 ratio = 4.40 and 15.96, respectively; *p*-value = 0.001).

Overall, the number of antigen-specific memory B-cells was significantly higher for all four antigens (PT, FHA, Prn, and tetanus) in both groups at 1 month and 1 year post-booster compared with pre-booster (Figure 4). However, the number of circulating memory B-cells 1 year post-booster had significantly declined in both groups.

### T-Cell Responses after a Preadolescent Tdap Booster Vaccination in Children 9 Years of Age

We found significantly lower IFN-γ levels in aP-primed compared with wP-primed children for FHA before the booster, for all four antigens (PT, FHA, Prn, and tetanus) at 1 month and for PT, FHA, and tetanus 1 year post-booster (Figure E2 in Supplementary Material), while IL-13 levels only differed for Prn before the booster vaccination between wP- and aP-primed children. Although IL-17 cytokine levels were significantly higher in wP-primed children for FHA and tetanus 1 month post-booster compared with aP-primed children, all IL-17 levels remained low, especially in relation to IFN-γ and IL-13 levels. The IL-10 cytokine levels of vaccine antigen-stimulated T-cells were low and still showed a high variability at all time points (Figure E2 in Supplementary Material).

**Figure 5:**
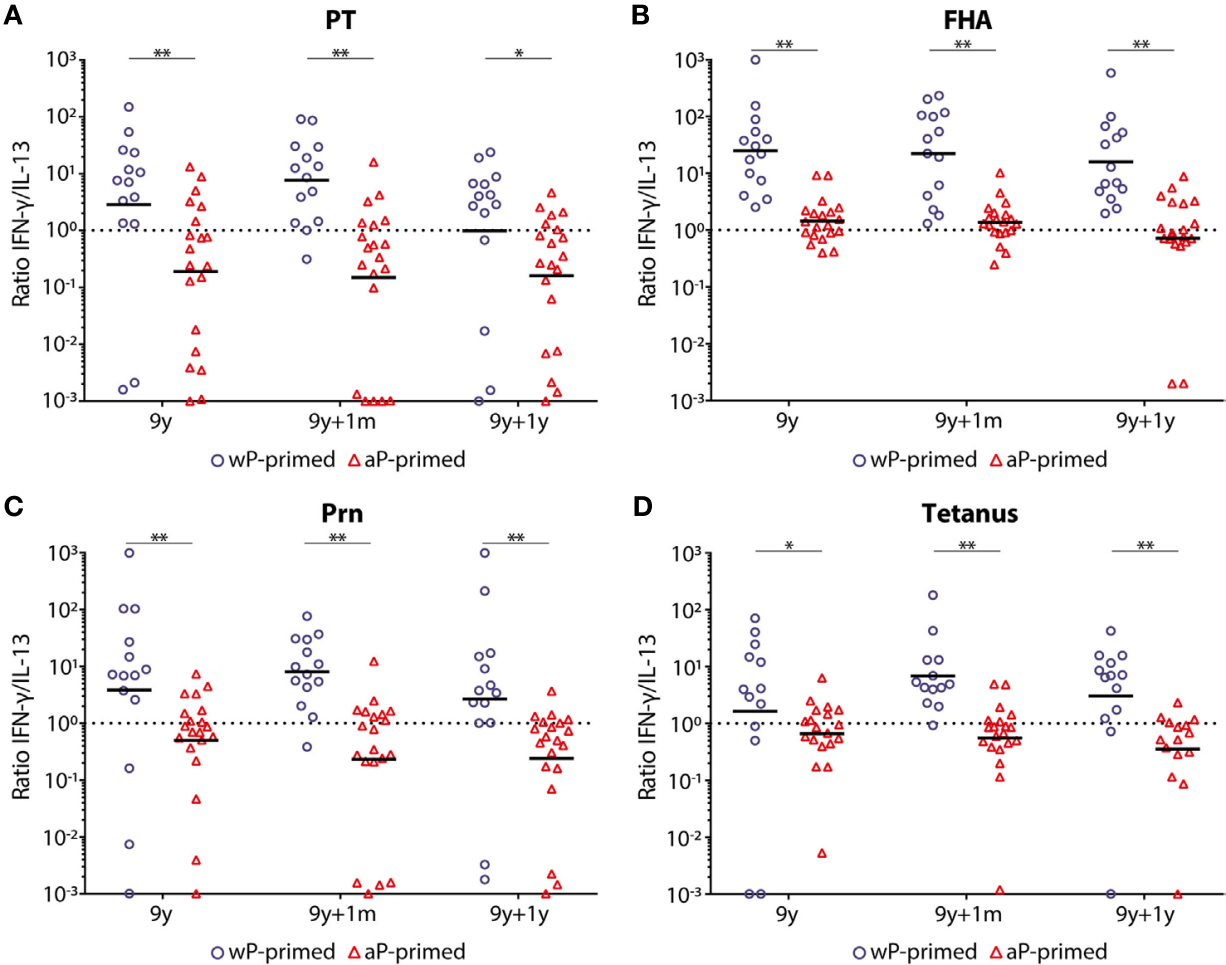
T-helper 1 (Th1)/Th2 ratio in children 9 years of age before and after a Tdap booster vaccination. The interferon-gamma (IFN-γ) (Th1)/interleukin-13 (IL-13) (Th2) ratio in supernatants of **(A)** pertussis toxin (PT), **(B)** filamentous hemagglutinin (FHA), **(C)** pertactin (Prn), or **(D)** tetanus toxoid-stimulated T-cells of whole-cell pertussis (wP)- (blue circles) and acellular pertussis (aP)-primed (red triangles) children 9 years of age, before, 1 month, and 1 year after a preadolescent Tdap booster vaccination. Note, black lines indicates geometric mean IFN-γ/IL-13 ratio and dotted lines indicates an IFN-γ/IL-13 ratio of 1. *p-Value <0.05 and **p-value <0.01; within the longitudinal study groups, there was only a significant difference for PT-stimulated T-cells between pre- and 1 year post-booster in wP-primed children (*p*-value <0.05).

With higher IFN-γ levels, the ratio of IFN-γ/IL-13 (Th1/Th2 cells) was >1 for all antigens and at all time points in wP-primed children (Figure 5), while this ratio was always <1 in aP-primed children, except for FHA both before and 1 month after the preadolescent booster. This resulted in significantly lower Th1/Th2 (IFN-γ/IL-13) ratios at all time points and for all antigens in aP-primed compared with wP-primed children (Figure 5).

No differences in IFN-γ, IL-13, IL-17, and IL-10 cytokine levels were observed between the time points pre- and post-booster within the wP- and aP-primed groups of children, except for lower PT-specific IL-10 production and FHA-specific IL-17 production in wP-primed children 1 year after the booster compared with 1 month (Figure E2 in Supplementary Material).

## Discussion

Based on epidemiological data, waning protection of pertussis-specific immunity seems to occur more rapidly in children who received aP vaccines at infant age in comparison with those who received wP vaccines. In this study, for the first time, we corroborate these epidemiological data with long-term immunological data in groups of children up to 10 years of age showing that priming during infancy with either wP or aP vaccines determines the humoral and cellular immune responsiveness to additional aP booster vaccinations until at least preadolescent age. We clearly showed that replacement of a DTwP combination vaccine by DTaP combination vaccines has enhanced pertussis immune responses on the short term till 6 years of age, 2 years after the fifth DTaP booster at preschool age. However, after the preadolescent Tdap booster vaccination, humoral and cellular immunity showed a shift. While Th2 responses were similar between the two groups, preadolescents primed with wP vaccines during infancy had the more favorable Th1-dominated immune response compared to aP-primed preadolescents for at least 1 year after the booster vaccination. This resulted in a Th2-skewed profile still present in adolescents primed with repeated aP vaccines during infancy that corroborates the increased pertussis incidence in aP-primed adolescents during pertussis outbreaks.

Although the resurgence of pertussis had already started before the introduction of aP vaccines, the incidence of pertussis has sharply increased since aP vaccines have been implemented in all primary and booster vaccination schedules in many countries worldwide (31). In the past years, the majority of the pertussis scientific community has reached consensus that priming with aP vaccines is less effective than with wP vaccines in maintaining pertussis immunity (31–33). It has been demonstrated that growing cohorts of older children and adolescents who only have received aP vaccines continue to be at higher risk of contracting pertussis than wP-primed individuals and thereby sustaining epidemics (34). In the Netherlands, the wP vaccines were replaced by aP vaccines in 2005. Therefore, we have not yet observed a difference in adolescent pertussis cases based on a different priming vaccine regime, as the fully aP vaccinated adolescents now are reaching the age of 13 years.

We showed that the aP-primed children 6 years of age had the advantage of having higher pertussis-specific antibody levels 2 years after the preschool booster compared with wP-primed children. This effect was lost at 9 years of age, with low antibody levels in both wP- and aP-primed children, although Prn-IgG levels remained higher in aP-primed children due to the much higher antibody response shortly after the preschool booster vaccination. Subsequently, the preadolescent booster vaccination induced lower pertussis-specific antibody levels and numbers of memory B-cells in aP-primed compared with wP-primed children. This implies that germinal center responses after wP-priming lead to a more sustainable memory B-cell compartment that is longer boostable during life. Interestingly, we showed that the rise in antibody levels upon a sixth aP vaccination was less than after the fifth aP vaccination, indicating that the degree of antibody production does not persist at the same level despite several vaccine doses. This might be explained by the lower pertussis antigen concentration in the preadolescent Tdap booster vaccination compared with the preschool DTaP booster vaccination. However, wP-primed children, vaccinated with the same DTaP and Tdap booster vaccines as the aP-primed children, showed higher pertussis-specific IgG levels 1 month after the preadolescent booster vaccination compared with 1 month after the preschool booster vaccination. This is in line with Eberhardt and Siegrist, who suggest that germinal center reactions induced by aP-priming vaccines are not effective enough to confer long-term memory responses (35). Furthermore, the more durable priming upon wP vaccination might especially be due to the presence of lipopolysaccharide, a highly immune-stimulatory bacterial cell wall component, operating as an adjuvant by activating innate immune cells *via* toll-like receptor 4 (36). The lack of immune-stimulatory components in the aP vaccines might explain the limited duration of effective immune responses against pertussis.

Importantly, the T-cell responses of aP vaccinated children showed a clear Th2-skewed profile, especially after the sixth aP vaccination at 9 years of age for the three pertussis vaccine antigens, as well as for the co-administered tetanus toxoid. Previous studies indicated more Th2 polarization in infants, children, and even in adults who have been primed with aP vaccines compared with wP-priming (37, 38). We now show that the polarization toward a Th2 profile in aP-primed preadolescents was caused by a decreased production of the Th1 cytokines (IFN-γ), and not by a higher Th2 cytokine (IL-13) production. Since Th1 and Th2 cytokine levels remained unchanged upon the preadolescent booster vaccination in both groups of children, Th1/Th2 ratios did not substantially change following the booster. This is in agreement with our earlier findings that aP-primed children 4 years of age showed high Th1 and Th2 responses, more effector memory and terminally differentiated CD4^+^ T-cells that remained unchanged or even decreased upon the preschool booster, whereas those in wP-primed children were low but increased upon a preschool booster vaccination (23, 39). Others also described that wP-primed adolescents showed less terminally differentiated pertussis-specific CD4^+^ T-cell responses than aP-primed adolescents (40). Natural boosting, due to the high circulation of pertussis, will enhance the vaccine induced T-cell responses during life. So, once a Th1 and/or a Th2 T-cell response has been induced by primary immunizations early in life, it most likely remains stable during life. Since Th1 cytokines play an important role in protection against pertussis (41), this might, at least in part, explain the better protection found in wP-primed individuals. Therefore, in future vaccine development strategies, it is advisable to change the Th2-skewed T-cell responses upon aP-priming in infancy into a more Th1-skewed response by introducing other adjuvants in the priming combination vaccines that stimulate the innate immune response toward a more Th1 profile (42). Another possible vaccination strategy could be to use a wP vaccine as a first priming vaccination to establish a Th1-dominated immune response, which is subsequently followed by aP vaccines to boost the Th1-primed immunity (41, 43).

Although Th17 responses have been reported to play an important role in protection against pertussis in mice and baboons (44, 45), we found just low Th17 responses in both wP- and aP-primed preadolescents, indicating that these responses may be far less pronounced upon vaccination in humans than in animal models. IL-10 might be involved in the regulatory T-cell response; however, we found similarly low values of IL-10 in both groups.

The recruitment of the two groups of 9-year-old preadolescents was conducted in two different years. This could introduce a different epidemiological background between the cohorts, given the frequent pertussis epidemics in the Netherlands. However, the proportion of PT-IgG seropositive participants [≥50 IU/mL as indication of recent infection (11)] was similar in the two groups. This indicates that the exposure to *B. pertussis* was most likely similar and therefore did not affect our study results.

In conclusion, our pertussis immune responses in preadolescents corroborate the epidemiological data showing that adolescents primed with aP vaccines are less protected against pertussis than those being primed with wP vaccines. New pertussis vaccines should be developed that induce a more Th1-dominated immune response in the primary vaccination series.

## Ethics Statement

This study was carried out in accordance with the recommendations of the medical research ethics committees united (MEC-U, Nieuwegein, the Netherlands) and registered at the European clinical trials database (2013-001864-50) and the Dutch trial register (www.trialregister.nl; NTR4089). For all subjects, both parents and/or legal representatives gave written informed consent in accordance with the Declaration of Helsinki. The protocol was approved by the MEC-U.

## Author Contributions

SL, LH, GB, and A-MB were involved in the conception, planning, study design, and participant enrollment. SL and LH performed laboratory analysis. SL performed statistical analysis. SL, ES, GB, and A-MB interpreted data and wrote the manuscript. All authors agreed to submit for publication.

## Conflict of Interest Statement

SL, LH, GB, and A-MB have no conflicts of interest. ES declares to have received grant support for vaccine studies from Pfizer and GSK.

## Footnotes

^1^https://eudract.ema.europa.eu.

^2^http://trialregister.nl.

## References

[1] Davis SF Strebel PM Cochi SL Zell ER Zell ER Hadler SC Pertussis surveillance – United States, 1989–1991. MMWR CDC Surveill Summ (1992) 41(8):11–9. 1491668

[2] van der Lee S Stoof SP van Ravenhorst MB van Gageldonk PGM van der Maas NAT Sanders EAM Enhanced *Bordetella pertussis* acquisition rate in adolescents during the 2012 epidemic in the Netherlands and evidence for prolonged antibody persistence after infection. Euro Surveill (2017) 22(47):17–00011. 10.2807/1560-7917.ES.2017.22.47.17-00011 29183555 PMC5710659

[3] Roehr B Whooping cough outbreak hits several US states. BMJ (2010) 341:c4627 10.1136/bmj.c4627 20736256

[4] Carcione D Regan AK Tracey L Mak DB Gibbs R Dowse GK The impact of parental postpartum pertussis vaccination on infection in infants: a population-based study of cocooning in Western Australia. Vaccine (2015) 33(42):5654–61. 10.1016/j.vaccine.2015.08.066 26320420

[5] Elomaa A He Q Minh NN Mertsola J. Pertussis before and after the introduction of acellular pertussis vaccines in Finland. Vaccine(2009) 27(40):5443–9. 10.1016/j.vaccine.2009.07.010 19628060

[6] Amirthalingam G Andrews N Campbell H Ribeiro S Kara E Donegan K Effectiveness of maternal pertussis vaccination in England: an observational study. Lancet (2014) 384(9953):1521–8. 10.1016/S0140-6736(14)60686-3 25037990

[7] Cherry JD Epidemic pertussis in 2012 – the resurgence of a vaccine-preventable disease. N Engl J Med (2012) 367(9):785–7. 10.1056/NEJMp1209051 22894554

[8] de Greeff SC Mooi FR Schellekens JF de Melker HE Impact of acellular pertussis preschool booster vaccination on disease burden of pertussis in the Netherlands. Pediatr Infect Dis J (2008) 27(3):218–23. 10.1097/INF.0b013e318161a2b9 18277916

[9] Black RE Cousens S Johnson HL Lawn JE Rudan I Bassani DG Global, regional, and national causes of child mortality in 2008: a systematic analysis. Lancet (2010) 375(9730):1969–87. 10.1016/S0140-6736(10)60549-1 20466419

[10] de Melker HE Versteegh FG Schellekens JF Teunis PF Kretzschmar M. The incidence of *Bordetella pertussis* infections estimated in the population from a combination of serological surveys. J Infect (2006) 53(2):106–13. 10.1016/j.jinf.2005.10.020 16352342

[11] de Greeff SC de Melker HE van Gageldonk PG Schellekens JF van der Klis FR Mollema L Seroprevalence of pertussis in the Netherlands: evidence for increased circulation of *Bordetella pertussis*. PLoS One (2010) 5(12):e14183. 10.1371/journal.pone.0014183 21152071 PMC2995730

[12] Lambert LC Pertussis vaccine trials in the 1990s. J Infect Dis (2014) 209(Suppl 1):S4–9. 10.1093/infdis/jit592 24626871 PMC3968808

[13] Clark TA. Changing pertussis epidemiology: everything old is new again. J Infect Dis (2014) 209(7):978–81. 10.1093/infdis/jiu001 24626532

[14] Klein NP Bartlett J Fireman B Baxter R. Waning Tdap effectiveness in adolescents. Pediatrics (2016) 137(3):e20153326. 10.1542/peds.2015-3326 26908667

[15] Sheridan SL Ware RS Grimwood K Lambert SB Number and order of whole cell pertussis vaccines in infancy and disease protection. JAMA (2012) 308(5):454–6. 10.1001/jama.2012.6364 22851107

[16] Witt MA Arias L Katz PH Truong ET Witt DJ. Reduced risk of pertussis among persons ever vaccinated with whole cell pertussis vaccine compared to recipients of acellular pertussis vaccines in a large US cohort. Clin Infect Dis (2013) 56(9):1248–54. 10.1093/cid/cit046 23487373

[17] Sheridan SL Ware RS Grimwood K Lambert SB. Reduced risk of pertussis in whole-cell compared to acellular vaccine recipients is not confounded by age or receipt of booster-doses. Vaccine (2015) 33(39):5027–30. 10.1016/j.vaccine.2015.08.021 26297874

[18] Warfel JM Zimmerman LI Merkel TJ. Acellular pertussis vaccines protect against disease but fail to prevent infection and transmission in a nonhuman primate model. Proc Natl Acad Sci U S A (2014) 111(2):787–92. 10.1073/pnas.1314688110 24277828 PMC3896208

[19] Berbers GA de Greeff SC Mooi FR Improving pertussis vaccination. Hum Vaccin (2009) 5(7):497–503. 10.4161/hv.8112 19242096

[20] Jackson DW Rohani P Perplexities of pertussis: recent global epidemiological trends and their potential causes. Epidemiol Infect (2013) 142(4):672–84. 10.1017/S0950268812003093 23324361 PMC9151176

[21] Hendrikx LH Berbers GA Veenhoven RH Sanders EA Buisman AM. IgG responses after booster vaccination with different pertussis vaccines in Dutch children 4 years of age: effect of vaccine antigen content. Vaccine (2009) 27(47):6530–6. 10.1016/j.vaccine.2009.08.052 19729085

[22] Hendrikx LH de Rond LG Oztürk K Veenhoven RH Sanders EA Berbers GA Impact of infant and preschool pertussis vaccinations on memory B-cell responses in children at 4 years of age. Vaccine (2011) 29(34):5725–30. 10.1016/j.vaccine.2011.05.094 21669247

[23] Schure RM Hendrikx LH de Rond LG Oztürk K Sanders EA Berbers GA T-cell responses before and after the fifth consecutive acellular pertussis vaccination in 4-year-old Dutch children. Clin Vaccine Immunol (2012) 19(11):1879–86. 10.1128/CVI.00277-12 23015649 PMC3491542

[24] Allen AC Mills KH. Improved pertussis vaccines based on adjuvants that induce cell-mediated immunity. Expert Rev Vaccines (2014) 13(10):1253–64. 10.1586/14760584.2014.936391 25017925

[25] Schure RM Hendrikx LH de Rond LG Oztürk K Sanders EA Berbers GA Differential T- and B-cell responses to pertussis in acellular vaccine-primed versus whole-cell vaccine-primed children 2 years after preschool acellular booster vaccination. Clin Vaccine Immunol (2013) 20(9):1388–95. 10.1128/CVI.00270-13 23825195 PMC3889587

[26] Hendrikx LH Felderhof MK Oztürk K de Rond LG van Houten MA Sanders EA Enhanced memory B-cell immune responses after a second acellular pertussis booster vaccination in children 9 years of age. Vaccine (2011) 30(1):51–8. 10.1016/j.vaccine.2011.10.048 22064265

[27] Schure RM de Rond L Oztürk K Hendrikx L Sanders E Berbers G Pertussis circulation has increased T-cell immunity during childhood more than a second acellular booster vaccination in Dutch children 9 years of age. PLoS One (2012) 7(7):e41928. 10.1371/journal.pone.0041928 22860033 PMC3409203

[28] Buisman AM de Rond CG Oztürk K Ten Hulscher HI van Binnendijk RS. Long-term presence of memory B-cells specific for different vaccine components. Vaccine (2009) 28(1):179–86. 10.1016/j.vaccine.2009.09.102 19799844

[29] van Gageldonk PG van Schaijk FG van der Klis FR Berbers GA. Development and validation of a multiplex immunoassay for the simultaneous determination of serum antibodies to *Bordetella pertussis*, diphtheria and tetanus. J Immunol Methods (2008) 335(1–2):79–89. 10.1016/j.jim.2008.02.018 18407287

[30] de Melker HE Versteegh FG Conyn-Van Spaendonck MA Elvers LH Berbers GA van Der Zee A Specificity and sensitivity of high levels of immunoglobulin G antibodies against pertussis toxin in a single serum sample for diagnosis of infection with *Bordetella pertussis*. J Clin Microbiol (2000) 38(2):800–6. 10655388 10.1128/jcm.38.2.800-806.2000 PMC86208

[31] Chen Z He Q. Immune persistence after pertussis vaccination. Hum Vaccin Immunother (2017) 13(4):744–56. 10.1080/21645515.2016.1259780 28045580 PMC5404361

[32] Carbonetti NH Wirsing von König CH Lan R Jacob-Dubuisson F Cotter PA Deora R Highlights of the 11th international *Bordetella* symposium: from basic biology to vaccine development. Clin Vaccine Immunol (2016) 23(11):842–50. 10.1128/CVI.00388-16 27655886 PMC5098017

[33] Bolotin S Harvill ET Crowcroft NS. What to do about pertussis vaccines? Linking what we know about pertussis vaccine effectiveness, immunology and disease transmission to create a better vaccine. Pathog Dis (2015) 73(8):ftv057. 10.1093/femspd/ftv057 26253079 PMC4626586

[34] Klein NP Bartlett J Rowhani-Rahbar A Fireman B Baxter R. Waning protection after fifth dose of acellular pertussis vaccine in children. N Engl J Med (2012) 367(11):1012–9. 10.1056/NEJMoa1200850 22970945

[35] Eberhardt CS Siegrist CA. What is wrong with pertussis vaccine immunity? Inducing and recalling vaccine-specific immunity. Cold Spring Harb Perspect Biol (2017) 9(12):a029629. 10.1101/cshperspect.a029629 28289058 PMC5710108

[36] Higgins SC Jarnicki AG Lavelle EC Mills KH. TLR4 mediates vaccine-induced protective cellular immunity to *Bordetella pertussis*: role of IL-17-producing T cells. J Immunol (2006) 177(11):7980–9. 10.4049/jimmunol.177.11.7980 17114471

[37] Dirix V Verscheure V Goetghebuer T Hainaut M Debrie AS Locht C Cytokine and antibody profiles in 1-year-old children vaccinated with either acellular or whole-cell pertussis vaccine during infancy. Vaccine (2009) 27(43):6042–7. 10.1016/j.vaccine.2009.07.075 19665604

[38] Bancroft T Dillon MB da Silva Antunes R Paul S Peters B Crotty S Th1 versus Th2 T cell polarization by whole-cell and acellular childhood pertussis vaccines persists upon re-immunization in adolescence and adulthood. Cell Immunol (2016) 30(4–305):35–43. 10.1016/j.cellimm.2016.05.002 27212461 PMC4899275

[39] de Rond L Schure RM Öztürk K Berbers G Sanders E van Twillert I Identification of pertussis-specific effector memory T cells in preschool children. Clin Vaccine Immunol (2015) 22(5):561–9. 10.1128/CVI.00695-14 25787136 PMC4412945

[40] Smits K Pottier G Smet J Dirix V Vermeulen F De Schutter I Different T cell memory in preadolescents after whole-cell or acellular pertussis vaccination. Vaccine (2013) 32(1):111–8. 10.1016/j.vaccine.2013.10.056 24176499

[41] Higgs R Higgins SC Ross PJ Mills KH. Immunity to the respiratory pathogen *Bordetella pertussis*. Mucosal Immunol (2012) 5(5):485–500. 10.1038/mi.2012.54 22718262

[42] Brummelman J Wilk MM Han WG van Els CA Mills KH. Roads to the development of improved pertussis vaccines paved by immunology. Pathog Dis (2015) 73(8):ftv067. 10.1093/femspd/ftv067 26347400 PMC4626578

[43] DeAngelis H Scarpino SV Fitzpatrick MC Galvani AP Althouse BM. Epidemiological and economic effects of priming with the whole-cell *Bordetella pertussis* vaccine. JAMA Pediatr (2016) 170(5):459–65. 10.1001/jamapediatrics.2016.0047 27018830 PMC6859645

[44] Mills KHG Gerdts V. Mouse and pig models for studies of natural and vaccine-induced immunity to *Bordetella pertussis*. J Infect Dis (2014) 209(Suppl 1):S16–9. 10.1093/infdis/jit488 24626866

[45] Warfel JM Merkel TJ. *Bordetella pertussis* infection induces a mucosal IL-17 response and long-lived Th17 and Th1 immune memory cells in nonhuman primates. Mucosal Immunol (2013) 6(4):787–96. 10.1038/mi.2012.117 23187316

