# Supplementary material for: Whole-Cell or Acellular Pertussis Primary Immunizations in Infancy Determines Adolescent Cellular Immune Profiles
Source: Front Immunol. 2018 Jan 24;9:51. doi: 10.3389/fimmu.2018.00051 (PMC5787539; doi:10.3389/fimmu.2018.00051)
Supplement: Supplementary file 1 [file data_sheet_1.docx]

**Recruitment procedures of children 9 years of age, primed with aP combination vaccines**

Children 9 years of age were recruited by sending invitation letters to the parents of eligible, healthy children being vaccinated according to the Dutch national immunization program (DTaP at 2, 3, 4, and 11 months of age and DTaP at 4 years of age). Exclusion criteria were: serious adverse event after previous vaccination; severe disease or medical treatment possibly interfering with study results as well as use of plasma products within 6 months, other vaccinations within a month; or antibiotic use/ fever within 14 days prior to enrolment. This study was approved by the medical research ethics committees united (MEC-U, Nieuwegein, the Netherlands), and registered at the European clinical trials database (2013-001864-50) and the Dutch trial register (www.trialregister.nl; NTR4089). For all participants, written informed consent was obtained from both parents or legal representatives. In September 2013, all children were enrolled in the study and received the Tdap booster vaccination according to study protocol.
