# Supplementary figures and images for: Whole-Cell or Acellular Pertussis Primary Immunizations in Infancy Determines Adolescent Cellular Immune Profiles
Source: Front Immunol. 2018 Jan 24;9:51. doi: 10.3389/fimmu.2018.00051 (PMC5787539; doi:10.3389/fimmu.2018.00051)

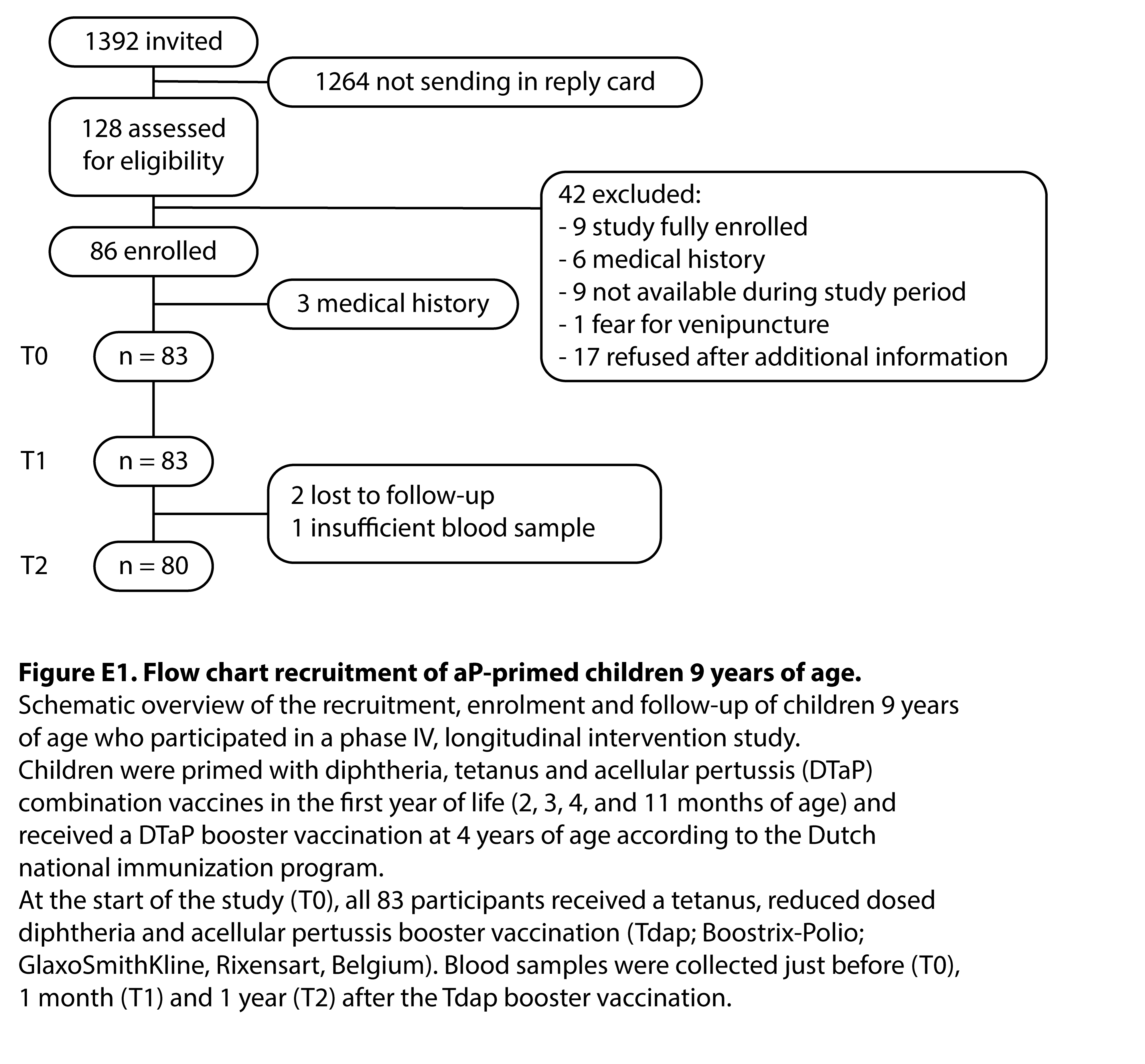

Supplement: Supplementary file 2 [file image_1.jpeg]

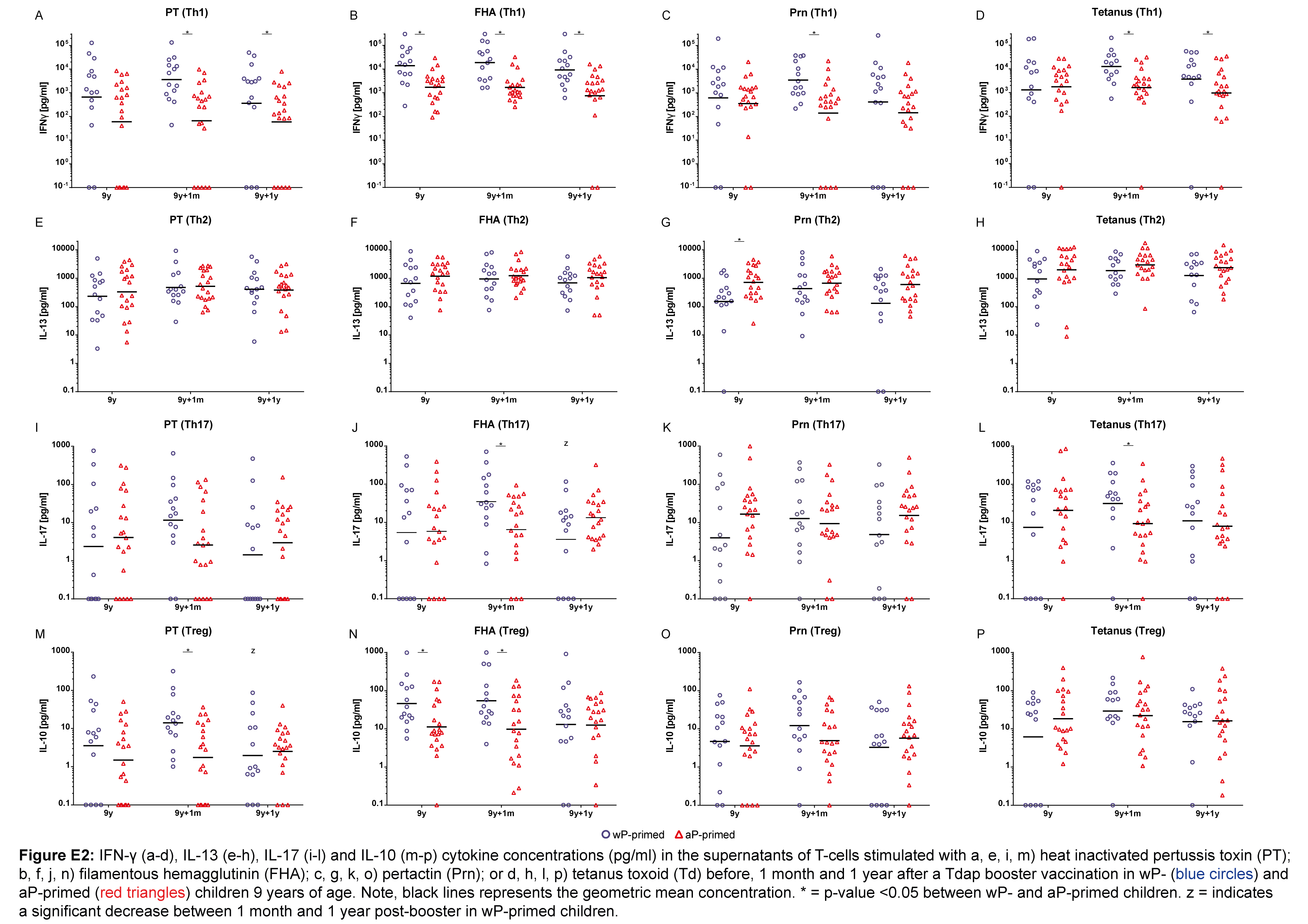

Supplement: Supplementary file 3 [file image_2.jpeg]
