# Supplementary material for: Whole-Cell or Acellular Pertussis Primary Immunizations in Infancy Determines Adolescent Cellular Immune Profiles
Source: Front Immunol. 2018 Jan 24;9:51. doi: 10.3389/fimmu.2018.00051 (PMC5787539; doi:10.3389/fimmu.2018.00051)
Supplement: Supplementary file 4 [file table_1.docx]

**Supplementary Table E1: IgG geometric mean concentrations (GMC) with corresponding 95% confidence intervals before and after a pre-school DTaP booster vaccination in children aged 4 years and before and after an additional Tdap booster vaccination in children aged 9 years**

|  |  | **wP-primed** | **aP-primed** | **p-value** |
| --- | --- | --- | --- | --- |
| **PT** IU/ml  (95%CI) | 4 years | 3.8 (2.8 - 5.1) | 6.5 (5.1 - 8.4) | **0.005** |
|  | 4 years + 1 month | 51.6 (34.9 - 76.1) | 163.5 (122.6 - 218.2) | **<0.001** |
|  | 6 years | 9.0 (6.6 - 12.2) | 21.6 (17.1 - 27.3) | **<0.001** |
|  | 9 years | 7.3^a^ (5.3 - 10.0) | 6.5 (4.8 - 8.8) | 0.635 |
|  | 9 years + 1 month | 112.4 (90.6 - 139.4) | 67.6 (56.6 - 80.7) | **<0.001** |
|  | 9 years + 1 year | 23.6 (18.3 - 30.5) | 18.7 (14.6 - 23.8) | 0.189 |
| **FHA** IU/ml  (95%CI) | 4 years | 7.4 (4.9 - 11.2) | 13.4 (10.3 - 17.5) | **0.016** |
|  | 4 years + 1 month | 161.5 (118.7 - 219.9) | 430.0 (356.4 - 518.7) | **<0.001** |
|  | 6 years | 37.3 (26.1 - 53.3) | 67.2 (55.1 - 81.9) | **0.005** |
|  | 9 years | 34.0^a^ (26.4 - 43.8) | 27.4^a^ (22.5 - 33.4) | 0.182 |
|  | 9 years + 1 month | 281.6 (244.0 - 325.1) | 158.1 (139.7 - 179.0) | **<0.001** |
|  | 9 years + 1 year | 105.5 (92.0 - 120.9) | 59.7 (50.1 - 71.1) | **<0.001** |
| **Prn** IU/ml  (95%CI) | 4 years | 3.2 (2.4 - 4.4) | 23.4 (18.3 - 29.9) | **<0.001** |
|  | 4 years + 1 month | 186.2 (129.4 - 267.9) | 1274.0 (932.6 – 1739.0) | **<0.001** |
|  | 6 years | 19.4 (13.3 - 28.4) | 121.3 (92.7 - 158.6) | **<0.001** |
|  | 9 years | 13.2^a^ (9.8 - 17.6) | 36.1^a^ (28.8 - 45.1) | **<0.001** |
|  | 9 years + 1 month | 343.3 (274.6 - 429.3) | 306.8 (267.2 - 352.4) | 0.394 |
|  | 9 years + 1 year | 93.1 (69.8 - 124.1) | 99.2 (83.8 - 117.5) | 0.703 |
| **Diphtheria** IU/ml (95%CI) | 4 years | 0.08 (0.07 - 0.11) | 0.08 (0.06 - 0.12) | 0.969 |
|  | 4 years + 1 month | 1.30 (1.00 - 1.69) | 0.82 (0.58 - 1.16) | **0.032** |
|  | 6 years | 0.18 (0.14 - 0.24) | 0.08 (0.06 - 0.11) | **<0.001** |
|  | 9 years | 0.05^b^ (0.04 - 0.06) | 0.04^b^ (0.03 - 0.05) | 0.119 |
|  | 9 years + 1 month | 0.91 (0.72 - 1.16) | 0.61 (0.49 - 0.75) | **0.012** |
|  | 9 years + 1 year | 0.19 (0.15 - 0.25) | 0.15 (0.12 - 0.19) | 0.170 |
| **Tetanus** IU/ml (95%CI) | 4 years | 0.24 (0.18 - 0.30) | 0.24 (0.19 - 0.31) | 0.826 |
|  | 4 years + 1 month | 8.88 (7.27 - 10.84) | 9.46 (7.59 - 11.79) | 0.671 |
|  | 6 years | 1.64 (1.28 - 2.11) | 1.20 (0.97 - 1.49) | 0.062 |
|  | 9 years | 0.47^a^ (0.39 - 0.56) | 0.35^a^ (0.29 - 0.44) | 0.053 |
|  | 9 years + 1 month | 9.24 (8.04 - 10.62) | 7.44 (6.31 - 8.76) | **0.046** |
|  | 9 years + 1 year | 2.08 (1.78 - 2.44) | 1.95 (1.65 - 2.30) | 0.550 |

a: GMC significant higher in 9-year-olds vs 4-year-olds (both before booster vaccination)

b: GMC significant lower in 9-year-olds vs 4-year-olds (both before booster vaccination)

Abbreviations: GMC: Geometric mean concentration; CI: confidence interval; wP: whole-cell pertussis vaccine; aP: acellular pertussis vaccine; PT: pertussis toxin; FHA: filamentous hemagglutinin; Prn: pertactin; IU: international units

Bold p-values represent values below 0.05.

Note: differences between pre and post IgG GMC’s within the longitudinal studies (pre and post 9-year-old booster vaccination samples) were tested with paired samples t-tests and were corrected for multiple comparison. For all antigens, GMC’s of pre vs 1 month, pre vs 1 year, and 1 month vs 1 year were all significantly different (p-values <0.001).
