# Supplementary material for: Whole-Cell or Acellular Pertussis Primary Immunizations in Infancy Determines Adolescent Cellular Immune Profiles
Source: Front Immunol. 2018 Jan 24;9:51. doi: 10.3389/fimmu.2018.00051 (PMC5787539; doi:10.3389/fimmu.2018.00051)
Supplement: Supplementary file 5 [file table_2.docx]

**Supplementary Table E2: Ratio of IgG antibody increase between before and 1 month after a Tdap booster vaccination at age 9 years. Children were primed with wP- or aP-combination vaccines in the first year of life (2, 3, 4, 11 months of age) and received a DTaP booster at age 4 years.**

|  |  | **wP-primed** | **aP-primed** | **p-value** |
| --- | --- | --- | --- | --- |
| **GM Ratio T1/T0** (95% CI) | PT | 15.1 (11.6 - 19.7) | 10.3 (8.2 - 13.0) | **0.031** |
|  | FHA | 8.1 (6.5 - 10.3) | 5.8 (4.9 - 6.8) | **0.016** |
|  | Prn | 25.0 (19.7 - 31.7) | 8.5 (7.1 - 10.2) | **<0.001** |
|  | Diphtheria | 18.6 (15.1 - 23.0) | 16.7 (13.5 - 20.8) | 0.481 |
|  | Tetanus | 19.8 (15.9 - 24.6) | 21.0 (17.2 - 25.8) | 0.690 |

Note: the GM ratio T1/T0 indicates level of increase between before and 1 month after vaccination at age 9 years. Differences between the groups were determined with independent samples t-tests, bold p-values indicate a significant difference between wP- versus aP-primed children.

Abbreviations: GM: geometric mean; CI: confidence interval; wP: whole-cell pertussis vaccine; aP: acellular pertussis vaccine; T0: before, T1: 1 month; PT: pertussis toxin; FHA: filamentous hemagglutinin; Prn: pertactin
